# Microbial Virulence Factors

**DOI:** 10.3390/ijms21155320

**Published:** 2020-07-27

**Authors:** Jorge H. Leitão

**Affiliations:** IBB–Institute for Bioengineering and Biosciences, Instituto Superior Técnico, Department of Bioengineering, Universidade de Lisboa. Av. Rovisco Pais, 1049-001 Lisboa, Portugal; jorgeleitao@tecnico.ulisboa.pt; Tel.: +351-21-841-7688

**Keywords:** microbial virulence factors, bacterial pathogens, fungal pathogens, pathogenicity

Microbial virulence factors encompass a wide range of molecules produced by pathogenic microorganisms, enhancing their ability to evade their host defenses and cause disease. This broad definition comprises secreted products such as toxins, enzymes, exopolysaccharides, as well as cell surface structures such as capsules, lipopolysaccharides, glyco- and lipoproteins. Intracellular changes in metabolic regulatory networks, governed by protein sensors/regulators and non-coding regulatory RNAs are also known to contribute to virulence. Furthermore, some secreted microbial products have the ability to enter the host cell and manipulate their machinery, contributing to the success of the infection. The knowledge, at the molecular level, of the biology of microbial pathogens and their virulence factors is central in the development of novel therapeutic molecules and strategies to combat microbial infections. This is of particular importance in the present days with the worldwide emergence of microbes resistant to available antimicrobials, as well as of novel pathogens such as the SARS-CoV-2 responsible for the present pandemics. Advances in recent years in molecular biology, genomics and post-genomics technologies, and bioinformatics contributed to the molecular identification and functional analyses of a wide range of microbial virulence factors. The Special Issue of IJMS focused on virulence factors and their regulatory networks from microbes such as bacteria, viruses, fungi, and parasites, as well as on the description of innovative experimental techniques to characterize microbial virulence factors. A total of 18 papers was published in this Special Issue. The collection comprises state of the art papers on virulence factors and mechanisms from a wide range of bacterial and fungal pathogens for humans, animals, and plants, thus reflecting the impact of microorganisms in health and economic human activities and the importance of the topic.

Due to their impact on human health, bacterial pathogens that cause infections in humans have received a higher attention, with *Escherichia coli* as one of the most studied bacteria. Pokharel et al. investigated the roles played by the recently described serine Protease Autotransporters (SPATE) TagB, TagC, and Sha of *E. coli* on urinary infections using a 5637 bladder epithelial cell line [1]. Members of the SPATE family owe their proteolytic activity to the serine protease catalytic triad composed of an aspartic acid, a serine, and a histidine residue. Evidence is presented showing that the three SPATE proteins are internalized by bladder epithelial cells, leading to alterations of actin cytoskeleton distribution. Results presented indicate that Sha and TagC degrade mucin and gelatin, respectively [1]. The mutation analysis of the serine catalytic site showed that secretion of the three proteins is not affected, but impaired their entry into epithelial cells, affecting their cytotoxicity and proteolytic activity [1].

The presence of genes related to virulence factors including adhesins, siderophores, protectines or invasins, and involved in allantoin metabolism were investigated among 32 non-*E. coli Enterobacterales* isolates obtained from the feces of 20 healthy adults [2]. Similar studies analyzed virulent NECE strains from patients with an ongoing infection, and not commensal NECE from healthy subjects as in the present study [2]. Isolates were taxonomically characterized by 16S RNA sequencing and MALDI-TOF MS analysis, and profiled by pulsed-field gel electrophoresis. The genus *Klebsiella* was found as the most represented, followed by *Enterobacter* and *Citrobacter* [2]. The isolates were further characterized concerning the presence in their genomes of genes encoding selected virulence factors, as well as their phenotypes related to biofilm formation and resistance to a selection of antibiotics. Results point out that the isolates do not encompass particularly virulent strains and in most of the cases were susceptible to antibiotics [2].

Yang et al. investigated the role of the *Salmonella enterica* serovar Typhimurium (ST) *pdxB*-*usg*-*truA*-*dedA* operon on intracellular survival using deletion mutants constructed with the λ-Red recombination technology [3]. The *Salmonella* genus comprises several facultative intracellular pathogens capable of infecting both human and animal hosts. The ST deletion mutants was investigated in J774A.1 macrophage cells. The deletion mutants Δ*pdxB*, Δ*usg*, and Δ*truA* exhibited reduced replication abilities compared to ST and the deletion mutant Δ*dedA*. The *pdxB-usg-truA-dedA* operon is shown to contribute to ST virulence in mice, and to resistance to oxidative stress [3].

*Aeromonas hydrophila* is an aquatic Gram-negative bacterium, capable of causing serious and lethal infections to a wide range of hosts, including fish, birds, amphibians, reptiles, and mammals [4]. Dong et al. described the identification and functional characterization of the LahS global regulator of *A. hydrophila* [4]. LahS was identified after the screening of a Tn5-derived library of 947 *A. hydrophila* mutants for reduced hemolytic activity. The LysR family transcriptional regulator family member LahS was found to play a role in biofilm formation, motility, antibacterial activity, resistance to oxidative stress, and proteolytic activity, as well as essential for *A. hydrophila* virulence to zebrafish [4]. The comparative proteomics analysis performed by the authors confirmed the role of the protein as a global regulator in *A. hydrophila* [4].

Bacteria of the *Dickeya* genus comprise plant pathogens that affect crops such as potatoes. In order to succeed when infecting their hosts, *Dickeya* secrete several proteins with plant cell wall degrading activities, including pectinases, cellulases, and proteases [5]. To investigate the role played by the protease Lon on *D. solani* pathogenicity towards potato, Figaj et al. used a λ-Red-derived protocol to construct a *lon* deletion mutant [5]. Results presented indicate that the Lon protein plays a role in protecting the bacterium to high ionic and temperature stresses, affecting the activity of pectate lyases, the organism motility, and delaying the onset of infection symptoms in the potato host [5].

The plant pathogen *Candidatus Phytoplasma mali* is the causal agent of apple proliferation disease, that affects apple production in Northern Italy [6]. *Phytoplasma* are biotrophic, obligate plant and insect bacterial symbionts, with a biphasic life cycle comprising reproduction in phloem-feeding insects and in plants [6]. The paper of Mittelberger et al. focused on the effector protein PME2 (Protein in Malus Expressed 2), expressed by *P. mali* when infecting apples [6]. The in silico analysis of the PME2 protein sequence performed revealed that the protein has features of effector proteins of Gram-positive bacteria, with a predicted final localization at the cytoplasm or nucleus of the host [6]. Two main protein variants, PME2ST PME2AT, were found associated in infected apple trees from Italy and Germany. Using protein variants tagged with GFP, both variants were found to translocate to the nucleus of *Nicotiana* spp. protoplasts. A better understanding of the molecular mechanisms used by *P. mali* to manipulate its host will rely on genomics analysis, since no genetic manipulation is presently available for these organisms [6].

The necrotrophic fungal pathogen *Sclerotinia sclerotiorum* (Lib.) de Bary infects a wide range of plants causing devastating agricultural losses. The organism forms a typical structure named sclerotia when vegetative hyphae gather to form a hardened multicellular structure important in its development and pathogenesis, and that under favorable conditions germinate leading to vegetative hyphae or apothecia that will initiate novel disease cycles by producing ascospores [7]. Li et al. used a proteomics approach based on 2D gels followed by spot isolation and protein identification by MALDI-TOF to identify proteins differentially expressed between a wild-type strain and a deletion mutant on the gene *SsNsd1* encoding a type IVb GATA zinc finger transcription factor [7]. Although the gene encoding SsNsd1 was found as expressed at low levels during the hyphae stage, the mutant is unable to form the compound appressoria. The authors were able to identify a total of 40 proteins as differentially expressed, 17 with predicted functions and 23 as hypothetical proteins [7]. The authors emphasize the utility of the approach used to identify important proteins involved in the SsNsd1-mediated formation of appressorium.

In addition to other factors, the success of pathogens rely on cell-cell communication. Bacterial outer membrane vesicles (OMV) are recognized as an efficient means of bacteria-bacteria and bacteria-host communication, not only intra-species, but also interspecies [8]. Despite the lack of data on a possible role played by OMVs in bacterial-yeast communication, Roszkowiak et al. investigated the role played by *Moraxella catarrhalis* OMVs on the susceptibility of selected bacterial and fungal pathogens to the cationic peptide polymyxin B, and to the serum complement [9]. Using OMVs from *M. catarrhalis* strain 6, the authors found that these OMVs conferred protection against the cationic peptide polymyxin B to the non-typeable *Haemophilus influenzae*, *Pseudomonas aeruginosa*, and *Acinetobacter baumannii*. Furthermore, OMVs also protected serum-sensitive non-typeable *H. influenza* and promoted the growth of the serum-resistant *P. aeruginosa* and *A. baumannii* against the complement [9]. In addition, the results presented also show that OMVs facilitate the formation of hyphae by the pathogenic yeast *Candida albicans*, promoting its virulence [9]. As stated by the authors, this work might pave the way to uncover additional roles played by OMVs-dependent interactions in multispecies communities [9].

The RNA chaperone Hfq is a master regulator of gene expression in bacteria, mediating the interaction of small noncoding RNAs with their mRNA targets, including those related to virulence in Gram-negative bacteria [10]. Dienstbier et al. performed an integrated Omics comparative analysis of the Hfq regulon in the *Bordetella pertussis* human pathogen, responsible for respiratory tract infections, in particular of a whooping cough [11]. Based on the use of RNAseq, and gene ontology analysis, genes significantly upregulated in the *hfq* mutant fall into categories including “Translation”, “Regulation of transcription”, and “Transmembrane transport”, while genes downregulated fall in the categories “Transmembrane transport”, “Iron–sulfur cluster assembly”, “Oxido-reduction process”, “Pathogenesis”, and “Protein secretion by the type III secretion system” [11]. Correlations of transcriptome, proteome, and secretome datasets are also presented [11]. Results presented corroborate the central role played by Hfq on the physiology and pathogenicity of *B. pertussis* [11].

In their brief report, Maisetta et al. performed the ex vivo evaluation of the bactericidal activity of combinations of the semi synthetic antimicrobial peptide lin-SB056-1 in combination with EDTA (Ethylenediaminetetraacetic acid) against endogenous *P. aeruginosa* present in the sputum from patients suffering from primary ciliary dyskinesia (PCD) [12]. The authors observed that the peptide and EDTA were almost inactive against PCD sputum endogenous *P. aeruginosa* when used alone, but exhibited a significant synergistic killing effect with a sputum sample-dependent efficacy [12]. EDTA, but not lin-SB056-1, was found to inhibit biofilm formation and the production of virulence factors including alginate, pyocyanin, and the metalloprotease LasA [12].

Various bacterial species have evolved various strategies to invade, survive, and multiply intracellularly in host cells. The paper of Denzer et al. presents an updated review of the mechanisms used by bacteria to invade the host cell, to manipulate their biochemical and gene expression machinery, and to multiply and escape from the host cell [13]. The authors present a thorough review of mechanisms used by intracellular pathogens, including the highjack of host immune defenses to enter into the host cell. Central attention is given to the various mechanism used to manipulate gene expression, including histone modification, control of host DNA methylation patterns, sabotage of host long non-coding RNAs, interfering with the host RNA transcription and translation, as well as with host protein stability [13]. The importance of the detailed molecular knowledge of pathogenesis mechanisms to the development of strategies to combat bacterial infections is highlighted [13].

The functions of grimelysin of *Serratia grimesii* and protealysin of *Serratia proteamaculans* that use actin as a substrate and promote bacterial invasion was reviewed by Khaitlina et al. [14]. The *Serratia* genus comprises facultative pathogens able to cause nosocomial infections or infections in immunocompromised patients, but nosocomial infections by *S. grimesii* or *S. proteamaculans* are low [14]. The paper focused on the discovery, properties and substrate specificity of the two proteases, their high specificity towards actin, and discussed their contribution to the invasiveness of *Serratia*, although further knowledge of the bacterium virulence factors and the cellular response mechanisms is required to fully understand the mechanism of *Serratia* invasion of the host cell [14].

The virulence factors that the bacteria use to cross the blood-brain barrier and cause meningitis is reviewed by Herold et al. [15]. Meningitis remains a worldwide problem often associated with fatalities and severe sequelae. After reviewing important traits of the central nervous system barriers to bacterial entrance, the authors review the various stages of the virulence processes of bacterial meningitis, including the processes of attachment and invasion, the routes used to enter the central nervous system, and the general mechanisms used to survive intracellularly [15]. The roles played by virulence factors produced by bacteria when crossing the central nervous system is also addressed, followed by the review of the specific traits of bacterial species more commonly associated with meningitis [15].

Coagulase-negative Staphylococci are a broad group of skin commensals that emerged as major nosocomial pathogens, with the species *S. epidermidis*, *S. haemolyticus*, *S. saprophyticus*, *S. capitis*, and *S. lugdunensis* as the most frequent pathogens [16]. In their paper, Argemi et al. reviewed the recent progress achieved in the pathogenomics of these species, based on published work supported by whole-genome data deposited in public databases [16]. As stated by the authors, the ever increasing amount of data available at the genomic, molecular, and clinical levels is expected to enhance the development of innovative approaches to characterize the pathogenicity of this bacterial group of pathogens [16].

Bacteria of the *Trueperella pyogenes* species are considered as belonging to the microbiota of animals skin and mucous membranes of the upper respiratory and urogenital tracts, but it is also an important opportunistic pathogen to animals, leading to important economic losses [17]. In their paper, Rzewuska et al. reviewed the taxonomy of the species, their pathogenicity to animals, and the various diseases associated, as well as their possible involvement in zoonotic infections, as well as the reservoirs and routes of transmission and infections [17]. The authors also present a thorough review of the main virulence factors used by the organism, including pyolysin, fimbriae, extracellular matrix-binding proteins, neuraminidases, and ability to form biofilms [17]. The availability of complete genome sequences and a better knowledge of *T. pyogenes* virulence factors, transmission routes, and epidemiology of infections is expected to lead to the development of effective vaccines, with particular hope deposited on DNA vaccines [17].

Candidiasis are on the rise worldwide, with *Candida albicans* and *Candida glabrata* as the more prevalent etiologic agents of these fungal diseases [18]. The paper by Galocha et al. thoroughly reviewed the distinct strategies used by the two *Candida* species to successfully cause human infections, starting by the adhesion and ability to form biofilms [18]. While *C. albicans* is dimorphic, growing as yeast or pseudohyphae, *C. glabrata* cannot undergo hyphal differentiation. As a consequence, *C. albicans* relies on the production of proteolytic enzymes and hyphal penetration to invade the host cell, while *C. glabrata* is thought to invade host cells by inducing endocytosis [18]. The authors extensively review the distinct mechanisms used by the two pathogenic to evade the host immune system, and succeed as pathogens. The detailed knowledge of the virulence mechanisms is critical to develop therapies that specifically target virulence traits of these two pathogenic yeasts [18].

Bacterial small non-coding regulatory RNAs (sRNAs) have emerged over the last decade as key regulators of post-transcriptional regulators of gene expression, being involved in a wide range of cellular processes, including bacterial virulence [19]. In their review, Pita et al. updated knowledge on sRNAs from two pathogens associated with respiratory infections and lung function decline of patients suffering from Cystic Fibrosis, *P. aeruginosa* and bacteria of the so-called *Burkholderia cepacia* complex (Bcc) [20]. As stated by the authors, the knowledge on *P. aeruginosa* sRNAs is far more extensive than from bacteria of the Bcc. After reviewing the main molecular characteristics of bacterial RNAs and their modes of action, including the role played by Hfq as a mediator of RNA-RNA interactions, the authors detail the description of the roles played by *P. aeruginosa* sRNAs known for their involvement in virulence traits of the bacterium. Despite the shorter information on Bcc sRNAs, the authors make a brief description of known sRNAs from Bcc [19]. The identification and functional characterization of additional sRNAs from these two pathogens will certainly enlighten our knowledge on their virulence traits.

The development of new tools to investigate microbial pathogenesis, at the molecular and cellular level, is of keen importance to comprehend how the microorganism can invade the host and cause infection. The paper from Hatlem et al. reviewed the basic molecular traits and applications of the SpyCatcher-SpyTag system, originally developed as a method for protein ligation [20]. The system consists of a modified domain of the SpyCatcher surface protein from *Streptococcus pyogenes* that recognizes the cognate SpyTag peptidic sequence composed of 13 amino acid residues [20]. Upon recognition, a covalent isopeptide bond is formed between a lysine side chain of the SpyCatcher and an aspartate of the SpyTag [20]. The authors describe in detail the fundamentals of the system and of related variants, emphasizing their uses in molecular studies of microbial virulence factors, surface proteins, membrane dynamics, as well as in the development of vaccines [20].

Microorganisms employ a wide array of virulence factors to successfully thrive and flourish with their hosts, leading this interaction to the development of infections that can often be fatal. The molecular knowledge of the virulence traits, associated with the recent availability of genomics data and bioinformatics tools for the more frequent human pathogens, is expected to lead in the near future of novel molecules and strategies to battle infectious diseases.

## References

[1] Pokharel P., Díaz J.M., Bessaiah H., Houle S., Guerrero-Barrera A.L., Dozois C.M. (2020). The Serine Protease Autotransporters TagB, TagC, and Sha from Extraintestinal Pathogenic *Escherichia coli* Are Internalized by Human Bladder Epithelial Cells and Cause Actin Cytoskeletal Disruption. Int. J. Mol. Sci..

[2] Amaretti A., Righini L., Candeliere F., Musmeci E., Bonvicini F., Gentilomi G.A., Rossi M., Raimondi S. (2020). Antibiotic Resistance, Virulence Factors, Phenotyping, and Genotyping of Non-*Escherichia coli Enterobacterales* from the Gut Microbiota of Healthy Subjects. Int. J. Mol. Sci..

[3] Yang X., Wang J., Feng Z., Zhang X., Wang X., Wu Q. (2019). Relation of the *pdxB-usg-truA-dedA* Operon and the *truA* Gene to the Intracellular Survival of *Salmonella enterica* Serovar Typhimurium. Int. J. Mol. Sci..

[4] Dong Y., Wang Y., Liu J., Ma S., Awan F., Lu C., Liu Y. (2018). Discovery of lahS as a Global Regulator of Environmental Adaptation and Virulence in *Aeromonas hydrophila*. Int. J. Mol. Sci..

[5] Figaj D., Czaplewska P., Przepióra T., Ambroziak P., Potrykus M., Skorko-Glonek J. (2020). Lon Protease Is Important for Growth under Stressful Conditions and Pathogenicity of the Phytopathogen, Bacterium *Dickeya solani*. Int. J. Mol. Sci..

[6] Mittelberger C., Stellmach H., Hause B., Kerschbamer C., Schlink K., Letschka T., Janik K. (2019). A Novel Effector Protein of Apple Proliferation Phytoplasma Disrupts Cell Integrity of *Nicotiana* spp. Protoplasts. Int. J. Mol. Sci..

[7] Li J., Zhang X., Li L., Liu J., Zhang Y., Pan H. (2018). Proteomics Analysis of SsNsd1-Mediated Compound Appressoria Formation in *Sclerotinia sclerotiorum*. Int. J. Mol. Sci..

[8] Toyofuku M., Nomura N., Eberl L. (2019). Types and origins of bacterial membrane vesicles. Nat. Rev. Microbiol..

[9] Roszkowiak J., Jajor P., Guła G., Gubernator J., Żak A., Drulis-Kawa Z., Augustyniak D. (2019). Interspecies Outer Membrane Vesicles (OMVs) Modulate the Sensitivity of Pathogenic Bacteria and Pathogenic Yeasts to Cationic Peptides and Serum Complement. Int. J. Mol. Sci..

[10] Feliciano J.R., Grilo A.M., Guerreiro S.I., Sousa S.A., Leitão J.H. (2016). Hfq: A Multifaceted RNA Chaperone Involved in Virulence. Future Microbiol..

[11] Dienstbier A., Amman F., Štipl D., Petráčková D., Večerek B. (2019). Comparative Integrated Omics Analysis of the Hfq Regulon in *Bordetella pertussis*. Int. J. Mol. Sci..

[12] Maisetta G., Grassi L., Esin S., Kaya E., Morelli A., Puppi D., Piras M., Chiellini F., Pifferi M., Batoni G. (2020). Targeting *Pseudomonas aeruginosa* in the Sputum of Primary Ciliary Dyskinesia Patients with a Combinatorial Strategy Having Antibacterial and Anti-Virulence Potential. Int. J. Mol. Sci..

[13] Denzer L., Schroten H., Schwerk C. (2020). From Gene to Protein—How Bacterial Virulence Factors Manipulate Host Gene Expression during Infection. Int. J. Mol. Sci..

[14] Khaitlina S., Bozhokina E., Tsaplina O., Efremova T. (2020). Bacterial Actin-Specific Endoproteases Grimelysin and Protealysin as Virulence Factors Contributing to the Invasive Activities of *Serratia*. Int. J. Mol. Sci..

[15] Herold R., Schroten H., Schwerk C. (2019). Virulence Factors of Meningitis-Causing Bacteria: Enabling Brain Entry across the Blood–Brain Barrier. Int. J. Mol. Sci..

[16] Argemi X., Hansmann Y., Prola K., Prévost G. (2019). Coagulase-Negative Staphylococci Pathogenomics. Int. J. Mol. Sci..

[17] Rzewuska M., Kwiecień E., Chrobak-Chmiel D., Kizerwetter-Świda M., Stefańska I., Gieryńska M. (2019). Pathogenicity and Virulence of *Trueperella pyogenes*: A Review. Int. J. Mol. Sci..

[18] Galocha M., Pais P., Cavalheiro M., Pereira D., Viana R., Teixeira M.C. (2019). Divergent Approaches to Virulence in *C. albicans* and *C. glabrata*: Two Sides of the Same Coin. Int. J. Mol. Sci..

[19] Pita T., Feliciano J.R., Leitão J.H. (2018). Small Noncoding Regulatory RNAs from *Pseudomonas aeruginosa* and *Burkholderia cepacia* Complex. Int. J. Mol. Sci..

[20] Hatlem D., Trunk T., Linke D., Leo J.C. (2019). Catching a SPY: Using the SpyCatcher-SpyTag and Related Systems for Labeling and Localizing Bacterial Proteins. Int. J. Mol. Sci..

